# Long Noncoding RNAs-Related Diseases, Cancers, and Drugs

**DOI:** 10.1155/2013/943539

**Published:** 2013-06-06

**Authors:** Jen-Yang Tang, Jin-Ching Lee, Yung-Ting Chang, Ming-Feng Hou, Hurng-Wern Huang, Chih-Chuang Liaw, Hsueh-Wei Chang

**Affiliations:** ^1^Department of Radiation Oncology, Faculty of Medicine, College of Medicine, Kaohsiung Medical University, Kaohsiung, Taiwan; ^2^Department of Radiation Oncology, Kaohsiung Medical University Hospital, Kaohsiung, Taiwan; ^3^Cancer Center, Kaohsiung Medical University Hospital, Kaohsiung Medical University, Kaohsiung, Taiwan; ^4^Department of Biotechnology, Kaohsiung Medical University, Kaohsiung, Taiwan; ^5^Doctor Degree Program in Marine Biotechnology, National Sun Yat-sen University/Academia Sinica, Kaohsiung, Taiwan; ^6^Institute of Clinical Medicine, Kaohsiung Medical University, Kaohsiung, Taiwan; ^7^Kaohsiung Municipal Ta-Tung Hospital, Kaohsiung, Taiwan; ^8^Institute of Biomedical Science, National Sun Yat-Sen University, Kaohsiung, Taiwan; ^9^Department of Marine Biotechnology and Resources, National Sun Yat-sen University, Kaohsiung, Taiwan; ^10^Graduate Institute of Natural Products, Kaohsiung Medical University, Kaohsiung, Taiwan; ^11^Department of Biomedical Science and Environmental Biology, Kaohsiung Medical University, Kaohsiung, Taiwan

## Abstract

Long noncoding RNA (lncRNA) function is described in terms of related gene expressions, diseases, and cancers as well as their polymorphisms. Potential modulators of lncRNA function, including clinical drugs, natural products, and derivatives, are discussed, and bioinformatic resources are summarized. The improving knowledge of the lncRNA regulatory network has implications not only in gene expression, diseases, and cancers, but also in the development of lncRNA-based pharmacology.

## 1. Introduction

Less than 2% of the mammalian genome is in protein-encoded regions, and the remainder is in noncoding RNAss (ncRNAs) [1]. Most long noncoding RNA (lncRNAs) are transcribed by RNA polymerase (Pol) II/Pol I, and some are transcribed by RNA Pol III [2]. The ncRNAs with nucleotide lengths of <200 and >200 are classified as short and long ncRNAs (lncRNAs), respectively. The lncRNAs can be further classified in terms of their orientation and location relative to neighboring genes as sense/antisense, divergent/convergent, and intronic/intergenic [3]. The lncRNAs function as chromatin scaffolds for complex assembly, as enhancers and decoys for improving and inhibiting transcription of target genes, and as *cis*-acting or *trans*-acting regulators of gene expression [4–6]. *Cis*-acting lncRNAs mediate local genes whereas *trans*-lncRNAs mediate multiple targets [6]. By dysregulating target gene expression, abnormal lncRNA expression causes cell dysfunction and disease progression. The official symbols of lncRNAs were designated by the HUGO Gene Nomenclature Committee [7].

## 2. The lncRNAs and Gene Expressions

The lncRNAs modulate cell cycle distribution and cell differentiation. For example, DNA damage-inducible lncRNA, namely, growth-arrested DNA damage-inducible gene 7 (gadd7), binds to TAR DNA-binding protein (TDP-43). By blocking the interaction between TDP-43 and cyclin-dependent kinase 6 (Cdk6) mRNA, gadd7 regulates cell cycle progression by promoting the decay of Cdk6 mRNA [17]. The lncRNAs reportedly modulate the differentiation of cells [18], the induction of pluripotent stem cell [19], and the induction of embryonic stem cells [20].

Some lncRNAs also have modulating effects on apoptosis [21]. For example, lncRNA, namely, erythroid prosurvival (EPS) is upregulated in terminal differentiation of murine erythroid cells [22] by inhibiting apoptosis [23]. Similarly, a study of melanoma cell lines showed that by downregulating sprouty homolog 4 intronic transcript 1 (SPRY4-IT1), lncRNAs inhibit cell proliferation and apoptosis [24].

In human cells, lncRNAs epigenetically regulate gene expression [25, 26] through chromatin remodeling [27]. For example, the mouse lncRNA, namely, potassium voltage-gated channel, KQT-like subfamily, member 1 (KCNQ1) overlapping transcript 1 (Kcnq1ot1) has a chromatin-interacting ability and can downregulate multiple genes in the Kcnq1 domain [28]. This gene silencing was reported to be mediated by DNA methylation at some target genes [29]. Other studies of cancer patients show that silenced tumor suppressor genes are often hypermethylated [30–32]. In the case of tumor suppressor genes, the epigenetic effect may have a role in carcinogenesis. In Hox antisense intergenic RNA (HOTAIR), long intergenic noncoding RNA (lincRNA), which is lncRNAs transcribed from noncoding DNA regions between protein-coding genes [33], may function as scaffolds for assembly of histone modification machinery [34].

Some lncRNAs may function through repeat sequences. For example, some lncRNAs that contain Alu elements [35] may transactivate Staufen 1- (STAU1-) mediated mRNA decay (SMD) by base pairing of Alu elements within both lncRNAs and 3′ untranslated region of the SMD target. These lncRNAs then downregulate several SMD targets [35]. 

## 3. The lncRNAs and Diseases

The functions of lncRNAs that are known to have roles in diseases have been reviewed previously [36, 37]. Recent studies suggest that lncRNAs have roles in neurodegenerative disorders [38, 39] and brain development [40]. In Huntington's disease, for example, neural lncRNAs are upregulated in taurine upregulated 1 (TUG1) and in nuclear paraspeckle assembly transcript 1 (NEAT1) but are downregulated in maternally expressed 3 (MEG3). The metastasis-associated lung adenocarcinoma transcript 1 (MALAT1) lncRNA is reportedly highly upregulated in neurons. In cultured hippocampal neurons, synaptic density is reduced by MALAT1 depletion but rescued by MALAT1 overexpression. Studies of patients with alcohol addiction reveal upregulated MALAT1 in the cerebellum, hippocampus, and brain stem [41], which suggests that the lncRNA network may have key roles in neurodegenerative processes [42].

Studies of patients with facioscapulohumeral muscular dystrophy (FSHD) involving Polycomb/Trithorax epigenetic regulation show a deregulated copy number in D4Z4 repeat mapping to 4q35 [43]. A recent study of FSHD patients further showed that selective upregulation of DBE-T, a chromatin-associated lncRNA, reverses repression of 4q35 gene transcription [44]. These results suggest that lncRNAs derived from repetitive sequences may contribute to disease development through epigenetic regulation.

Recently, the single nucleotide polymorphisms (SNPs) of lncRNAs have been found to play important roles for disease association studies. For example, the SNP rs1333049 in the lncRNA, namely, antisense noncoding RNA in the INK4 locus (ANRIL) is reportedly associated with myocardial infarction as well as the pharmacogenomic evaluation in hypercholesterolemia [45]. SNP rs2383207 on lncRNA-ANRIL and SNP rs11066001 on protein-coded BRCA1 associated-protein (BRAP) gene were both associated with ankle-brachial index in a Taiwanese population [46]. Three SNPs (rs2067051, rs2251375, and rs4929984) located in 5′ region of the H19 imprinted maternally expressed transcript (H19) genes were reportedly associated with birth weight [47]. Additionally, the rs2839698 TC genotype of H19 was reportedly associated with a low risk for nonmuscle-invasive disease [48].

## 4. The lncRNAs and Cancers

Aberrant lncRNA expression contributes to tumor development in many cancer types [49–55]. For example, an lncRNA microarray showed that some lncRNAs contribute to glioma carcinogenesis [56, 57]. The lncRNAs also have important roles in the development of lung [58], breast [59], and liver cancers [60].

 The accumulating evidence of lncRNA involvement in carcinogenesis includes findings that downregulation of maternally expressed gene 3 (MEG3), an imprinted lncRNA, is associated with carcinogenesis of meningiomas [61] and bladder cancer [62]. The lncRNA, namely, ANRIL also contributes to the development of plexiform neurofibromas in neurofibromatosis type 1 [63]. The ANRIL downregulates tumor suppressor gene p15 (INK4B) expression by binding to and recruiting the suppressor of zeste 12 homolog (*Drosophila*) (SUZ12), a component of the Polycomb Repressive Complex 2 [64]. When DNA damage occurs, ANRIL is upregulated by the ATM-E2F1 signaling pathway [65].

In human colorectal cancer, lncRNA H19 and H19-derived miR-675 are overexpressed in cell lines and primary tissues but not in adjacent noncancerous tissues [66]. Exogenous miR-675 expression also downregulates the tumor suppressor retinoblastoma, which is a direct target of miR-675 and increases tumor cell growth. Upregulation of H19 is also known to contribute to gastric cancer cell proliferation [67] and bladder cancer metastasis [68].

 The HOTAIR is overexpressed in breast [69], nasopharyngeal [70], and liver [71] cancers. Loss of HOTAIR moderates the invasiveness of breast cancer, particularly in cells with upregulated Polycomb Repressive Complex 2 (PRC2). In nasopharyngeal and hepatocellular carcinoma, upregulated expression of HOTAIR indicates a poor prognosis [70, 71].

In lung cancer cells, downregulation of MALAT1 by siRNA decreases cell motility and downregulates motility-related genes [72], which suggests that MALAT1 promotes lung cancer metastasis. Similarly, MALAT1 is important in regulating cell proliferation, migration, and invasion of colorectal cancer metastasis [73]. In bladder cancer tissues, MALAT1 is overexpressed. Downregulation of MALAT1 by siRNA, the epithelial-to-mesenchymal transition-related genes, and cell migration of bladder cancer cells are inhibited [74]. After liver transplantation, MALAT1 is overexpressed in both cell lines and tissues of patients with hepatocellular carcinoma. Additionally, upregulated MALAT1 is associated with increased risk of liver tumor recurrence [75].

An lncRNA of highly upregulated liver cancer (HULC) is reportedly overexpressed in hepatocellular carcinoma [76]. The HULC may downregulate miR-372 and induce phosphorylation of cAMP responsive element binding protein 1 (CREB1) in liver cancer [77]. Similarly, overexpressed lncRNA, namely, urothelial carcinoma associated 1 (UCA1) affects cell proliferation and invasion in bladder cancer [78]. The CREB1 is involved in the UCA1-mediated cell cycle distribution of bladder cancer [79]. Another lncRNA, UCA1a (cancer-upregulated drug-resistant gene, CUDR), reportedly regulates the carcinogenesis of human bladder cancer [80].

Methylation may also have a modulating role in lncRNA expression. For example, a study of triple-negative breast cancer cell lines showed hypermethylation and downregulation in both miR-31 and its MIR31 host gene (MIR31HG) [81]. The lncRNA, namely, colorectal neoplasia differentially expressed (CRNDE) is overexpressed in colorectal cancer and leukemia [82]. In esophageal adenocarcinoma, high-resolution methylome analyses have shown hypomethylated noncoding DNA regions and upregulated lncRNA in actin filament-associated protein 1 (AFAP1) antisense RNA 1 (AFAP1-AS1) [83].

Similar to the disease association studies as described above, the accumulating evidence of SNPs in lncRNAs has been reported in cancer association studies. For example, SNP array-based study reported that several SNPs in lncRNAs were associated with prostate cancer risk [84]. An lncRNA prostate cancer gene expression marker 1 (PCGEM1) is overexpressed in prostate cancer [85]. Two tagSNPs (rs6434568 and rs16834898) of the PCGEM1 were reported to be associated with prostate cancer [86]. Several lncRNAs contain SNPs such as rs7763881 in highly upregulated in liver cancer long noncoding RNA (HULC) and rs619586 in MALAT1 which are reportedly associated with decreased hepatocellular carcinoma risk [87].

## 5. The lncRNAs and Their Potential Modulators

Chemically engineered oligonucleotides that have proven effectivess for targeting endogenous miRNAs in mice [88] have potential applications in lncRNAs. For example, antisense oligonucleotides targeted at the mouse lncRNA Malat1 correct RNA gain-of-function effects of myotonic dystrophy [89]. Using siRNA treatment to lncRNA, the lncRNA, namely, antidifferentiation ncRNA (ANCR) is downregulated to promote osteoblast differentiation [90]. Similarly, siRNA-based downregulation of lncRNA associated with liver regeneration (LALR1) inhibits hepatocyte proliferation and cell cycle progression during liver regeneration [91]. Data obtained by a recent systematic transcriptome-wide analysis of lncRNA-miRNA interactions [92] may reveal additional regulators of lncRNA expression such as miRNAs that contribute to lncRNA degeneration. For example, in some lncRNAs targeted by breast cancer-related miRNAs, changes in gene expressions differ between women with and without breast tumors [93].

Inhibitors that modulate lncRNA function have also been identified. For example, small molecules such as diazobenzene-related compounds are now known to inhibit the function of miR-21 [94], a polyadenylated lncRNA [95]. 5-aza-2′-deoxycytidine (5-aza-dC), a methylation inhibitor, inhibits the methylation of putative imprinted control region (ICR) of H19 gene and leads to the downregulation of the H19 mRNA expression in blastocysts derived from vitrified two-cell embryos [96]. This finding suggests that epigenetic agents may be the modulators for lncRNA expression as well as their related targeting signals.

The hypothesis that environmental exposures are another cause of ncRNA alterations [97] was tested by exposing aquatic midges to xenobiotics, which revealed upregulation of lncRNAs derived from repetitive sequences [98]. Additionally, telomeric and centromeric ncRNA can be activated by bisphenol A, a synthetic chemical with estrogen-like effects [98]. Based on these findings, some drugs may also modulate lncRNA expression. Therefore, many natural products and their derivatives are likely to prove suitable for screening and identifying these modulators in lncRNAs.

## 6. Long Noncoding RNA and Bioinformatics Resources

Computational methods for predicting lncRNA function have been well reviewed [99]. Recently, consistently improving computational capability enabled rapid development of functional analyses and bioinformatics resources for lncRNAs [100]. Except for NRED [8], ncFANs [9], and lncRNAdb [10], we summarize the update progression of bioinformatics resources for lncRNAs during 2012-2013 as shown in Table 1.

For example, the NRED [8] database of lncRNA expression includes both microarray and *in situ* hybridization data for human and mouse lncRNAs. The noncoding RNA Function Anotation server (ncFANs) [9], a web server for functional anotation of lncRNAs, includes ten reannotated human and mouse microarray datasets. The lncRNAdb [10] is a comprehensive database of eukaryotic lncRNA anotations. The data contained in the lncRNAdb include sequences, structures, genomic contexts, expressions, and subcellular distributions. Most (~75%) lncRNAs in the database were collected from mammals.

The NONCODE v3.0 [11] is an integrated database of lncRNA anotations obtained from re-annotated and updated microarray data from NONCODE v2.0 [101]. The NONCODE v3.0 database includes a visualized Genome Browser and a BLAST-based sequence alignment search. Since the secondary structure of an lncRNA may affect its protein interactions, the LNCipedia [12] provides helpful information for visualizing the structures of annotated lncRNA sequences. The LNCipedia also uses an algorithm for predicting potential coding scores for each transcript and an HMMER algorithm for searching for RNA sequences in Pfam protein domains. The LncRNADisease [102] provides experimentally validated lncRNA—disease associations for 166 diseases in curated lncRNA interacting partners at the protein, RNA, miRNA, and DNA levels. Similarly, DIANA-LncBase provides experimentally verified and computationally predicted miRNA target sites of human and mouse lncRNAs [14]. The iSeeRNA [15] webserver was constructed by using a support vector machine- (SVM-) based classifier to identify lincRNAs from transcriptome sequencing data. Based on next-generation sequencing (ChIP-Seq) data, ChIPBase [16] provides anotations and identifyies information for transcription factor binding sites (TFBS) of lncRNAs and miRNAs from chromatin immunoprecipitation. A database of the regulatory relationships of transcription factors/lncRNA and transcription factors/miRNA is also being considered.

## 7. Conclusion

Various lncRNA functions are essential for regulating gene expression. This study focused on lncRNA dysregulation associated with disease progression and carcinogenesis and on the development of drugs for modulating lncRNA function. Since lncRNA is rarely studied in natural products, the resources mentioned in the paper may provide helpful information for researchers studying natural products.

## Figures and Tables

**Table 1:** The lncRNA bioinformatics resources.

Name	Description
NRED [8]	Database of lncRNA expression. (http://nred.matticklab.com/cgi-bin/ncrnadb.pl/)
ncFANs [9]	Web server for functional annotation of lncRNAs. (http://ebiomed.org/ncfans/)
lncRNAdb [10]	Database of comprehensive annotations of functional lncRNAs. (http://www.lncrnadb.org/)
NONCODE [11]	Database of integrative annotations of lncRNAs. (http://www.noncode.org/NONCODERv3/guide.htm)
LNCipedia [12]	Database of annotations and structures of lncRNA sequences. (http://www.lncipedia.org/)
LncRNADisease [13]	Database of lncRNA-associated diseases. (http://cmbi.bjmu.edu.cn/lncrnadisease/)
DIANA-LncBase [14]	Database of microRNA targets on lncRNAs. (http://www.microrna.gr/LncBase/)
iSeeRNA [15]	The lincRNA transcripts identified from transcriptome sequencing data. (http://www.myogenesisdb.org/iSeeRNA/)
ChIPBase [16]	Database for annotating and exploring the expression profiles in transcriptional regulation of lncRNAs and other ncRNAs. (http://deepbase.sysu.edu.cn/chipbase/)
